# Grazing-incidence small-angle X-ray scattering study of correlated lateral density fluctuations in W/Si multilayers

**DOI:** 10.1107/S2053273318017382

**Published:** 2019-02-12

**Authors:** K. V. Nikolaev, S. N. Yakunin, I. A. Makhotkin, J. de la Rie, R. V. Medvedev, A. V. Rogachev, I. N. Trunckin, A. L. Vasiliev, C. P. Hendrikx, M. Gateshki, R. W. E. van de Kruijs, F. Bijkerk

**Affiliations:** a MESA+ Institute for Nanotechnology, University of Twente, Netherlands; b NRC Kurchatov Institute, Moscow, Russia; c Malvern Panalytical B.V., Almelo, Netherlands

**Keywords:** diffuse scattering, density fluctuations, multilayer coatings, thin films

## Abstract

A structural characterization of W/Si multilayers using X-ray reflectivity (XRR), scanning transmission electron microscopy (STEM) and grazing-incidence small-angle X-ray scattering (GISAXS) is presented. STEM images revealed lateral, periodic density fluctuations in the Si layers, which were further analysed using GISAXS. Characteristic parameters of the fluctuations such as average distance between neighbouring fluctuations, average size and lateral distribution of their position were obtained by fitting numerical simulations to the measured scattering images, and these parameters are in good agreement with the STEM observations. For the numerical simulations the density fluctuations were approximated as a set of spheroids distributed inside the Si layers as a 3D para­crystal (a lattice of spheroids with short-range ordering but lacking any long-range order). From GISAXS, the density of the material inside the density fluctuations is calculated to be 2.07 g cm^−3^ which is 89% of the bulk value of the deposited layer (2.33 g cm^−3^).

## Abstract 2

An inhomogeneity of material in W/Si multilayer structures was studied with grazing-incidence small-angle X-ray scattering. The experimental study revealed lateral density fluctuations in the Si spacer layers.

## 1. Introduction   

Thin film periodic multilayer reflective coatings are used in spectroscopy and optical instruments in the photon energy range of a few tens of eV up to a few keV, *i.e.* from the extreme UV (XUV) to the soft X-ray range (Fewster, 1996 ▸; Louis *et al.*, 2011 ▸). Depending on the application, various aspects of the structural quality of reflective coatings should be prioritized during the coating development process. As an example, often the reflectivity should be maximized over a certain bandwidth around a particular wavelength. However, for some applications like X-ray focusing optics the minimization of the off-specular diffuse scattering can be more important than maximization of the reflectivity. Primarily, diffuse scattering in multilayers is caused by interface roughness. In specific cases, diffuse scattering in multilayers may also be caused by a 3D distribution of defects in the volume of the structure (Pietsch *et al.*, 2013 ▸). Analysis of the diffuse scattering provides information about the growth process including defects (Siffalovic *et al.*, 2011 ▸). Such information can indicate directions for further optimization of the reflective properties of the multilayers.

In this study we focus on the characterization of the structural imperfections of a W/Si periodic multilayer with a 4.5 nm period. Such a multilayer is typically used for fluorescent spectroscopy analysis in the XUV range. A first multilayer structure characterization using scanning transmission electron microscopy (STEM) suggested that the Si spacer layers have lateral density fluctuations, different to what was expected from the growth model (Pelliccione & Lu, 2008 ▸). This observation was confirmed using grazing-incidence small-angle X-ray scattering (GISAXS). Numerical simulations of the GISAXS experimental data were performed to analyse the statistics of the distribution of the density fluctuations and the morphology of the interface roughness.

## 2. Sample preparation and characterization   

### 2.1. Sample preparation   

W/Si multilayers were deposited on Si super-polished substrates using a magnetron sputtering system (Louis *et al.*, 2011 ▸). A 6.35 mm-thick substrate was used in order to avoid a deformation (curvature) of the substrate caused by the tensile stress in the multilayer. A W target of 99.95% and Si target of 99.99% purity were used. Ar was used as a sputtering gas at a base pressure of 0.01 Pa. Magnetrons were set at a constant power mode equal to 86.7 and 213 W for the W and Si targets, respectively. The spatial uniformity of the coating was achieved by rotating the sample holder at 60 r min^−1^ during the deposition. The multilayer coating prepared contained 50 W/Si bilayers with a period thickness *D* = 4.5 nm and W layer thickness to period thickness ratio of 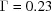.

### 2.2. Preliminary sample characterization with XRR and HAADF-STEM   

As a post-growth characterization of the manufactured sample, X-ray reflectivity (XRR) was used. The XRR curve was measured on an Empyrean laboratory diffractometer from Malvern Panalytical using characteristic Cu *K*α_1_ radiation from a long fine-focus line source. Monochromatization and primary collimation of the incident beam were achieved using a four-bounce asymmetrically cut germanium monochromator, which yields a beam divergence of 0.012°. Collimation at the detector side was achieved by an anti-scatter slit in combination with a tunable electronic receiving slit of a PIXcel3D detector. XRR data were analysed using the free-form approach (Zameshin *et al.*, 2016 ▸). The resulting electron-density depth profile was used to verify initial estimations of *D* and Γ, resulting in *D* = 4.46 nm and Γ = 0.2. The reconstructed parameters differ slightly from the design values because of the inter-diffusion between W and Si occurring during the deposition. These parameters are used further for the GISAXS numerical simulations presented in Section 4 for the qualitative investigation of the structure of the Si layer. XRR experimental data and analysis are presented in Appendix *B*2.

STEM measurements were performed in a Titan 80-300 equipped with a spherical aberration corrector (probe corrector), using an acceleration voltage of 300 kV. The microscope was equipped with an energy-dispersive X-ray Si(Li) spectrometer (EDAX), high-angle annular dark-field electron detector (Fischione) and Gatan image filter.

High-resolution bright-field transmission electron microscopy (TEM) measurements and an electron diffraction pattern (not shown here) indicated the presence of W nanocrystals in the W layers, with a crystallite size comparable with the W layer thickness, typical for nanometre-thickness metal layers grown by sputter deposition.

**Figure 1:**
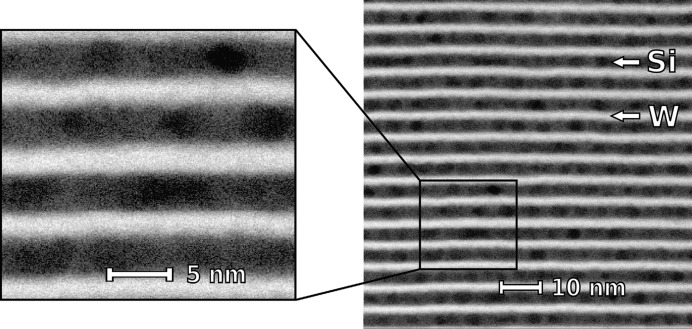
HAADF-STEM image of a W/Si multilayer. The W layers are shown as brighter areas and Si as darker areas.

To study the microstructure of the Si layers, high-angle annular dark-field scanning transmission electron microscopy (HAADF-STEM) measurements were performed. The HAADF-STEM images (see Fig. 1 ▸) revealed the presence of inhomogeneity inside the Si layers. Such inhomogeneity is unexpected, since to our knowledge amorphous Si is not known to develop any such density fluctuations during growth.

In Fig. 1 ▸ the Si layers appear darker than the W layers due to both atomic number contrast in HAADF-STEM and thickness effects as considered by Van den Broek *et al.* (2012 ▸). The inhomogeneity inside the Si layers appears to be quasi-periodic, and its distribution is statistically analysed in Appendix *B*1. The HAADF-STEM image, however, does not provide more detailed information on the density fluctuations including their positional correlations throughout the multilayer. To extract such information, a GISAXS study was performed, since GISAXS is highly sensitive to correlated structural imperfections [see the comprehensive review by Renaud *et al.* (2009 ▸)].

### 2.3. GISAXS experiment   

GISAXS measurements were done on the bending-magnet beamline Langmuir of the synchrotron radiation source Siberia-2 at the Kurchatov Institute (Korchuganov *et al.*, 2012 ▸). Monochromatization at the beamline is carried out by a thermally stabilized two-bounce Si monochromator with (111) reflection. Higher harmonics of the monochromated beam are suppressed with quartz and tungsten X-ray mirrors. The synchrotron beam was collimated with three sets of slits. The resulting beam size is 50 × 300 µm and the corresponding average direct-beam intensity is approximately 3 × 10^7^ counts s^−1^. The vertical beam divergence is 4 arcsec and horizontal beam divergence 20 arcsec.

**Figure 2:**
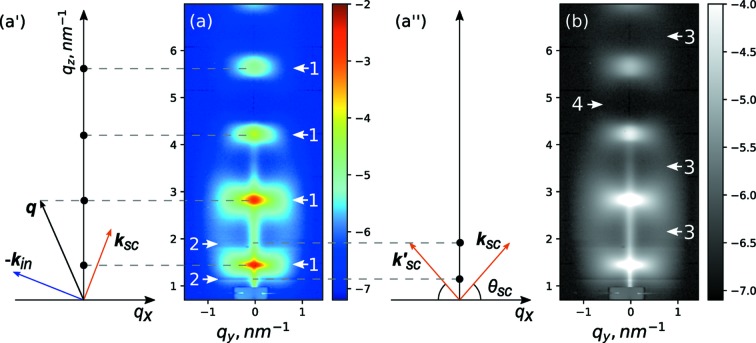
(*a*) Experimentally measured GISAXS intensity in arbitrary units on a logarithmic scale. (*b*) The same experimental data, though the colour scheme and colour depth are chosen to emphasize low-intensity features. (*a*′) Scattering geometry for the Bragg sheets. (*a*′′) Scattering geometry for the Bragg singularity lines.

Experimental data were measured with a Pilatus 100k 2D detector. Measurements were taken at the wavelength λ = 0.1 nm in 12 exposures of 15 min each, in order to avoid detector saturation. The angle of incidence was set to 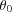 = 0.4°, in between total external reflection and the first Bragg peak. The sum of 12 GISAXS measurements is shown in Fig. 2 ▸ on a logarithmic scale, where the colour scale of Fig. 2 ▸(*a*) is chosen to emphasize high-intensity scattering, whereas the same data are given in Fig. 2 ▸(*b*) with the colour scale adjusted to emphasize lower-intensity details.

The most pronounced feature in the GISAXS scattering is the resonant diffuse scattering sheets (Kaganer *et al.*, 1996 ▸). The series of these sheets is marked by arrows ‘1’ in Fig. 2 ▸(*a*). These sheets are caused by the correlated interface roughness (Holý & Baumbach, 1994 ▸). In the literature, resonant diffuse scattering sheets are also referred to as Bragg sheets (Siffalovic *et al.*, 2009 ▸). The scattering geometry for the Bragg sheets is shown in Fig. 2 ▸(*a*′). Here, 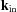, 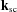 are the wavevectors for the incident and scattered beam, respectively, and 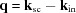 is the reciprocal-space vector. Bragg sheets are located at 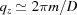.

A second feature marked by arrows ‘2’ in Fig. 2 ▸(*a*) is known in the literature as the Bragg singularity lines (Kaganer *et al.*, 1995 ▸). These Bragg singularity lines are minima caused by the destructive interference of the scattered waves with each other [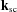 and 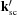 in Fig. 2 ▸(*a*′′)], while Bragg sheets are due to the Bragg interference of incident and scattering waves. Bragg singularity lines are located at positions for which the exit angle of the scattered beam 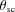 satisfies the Bragg condition 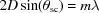 for the multilayer structure.

In addition to the features discussed above one can observe two other features in Fig. 2 ▸(*b*). The first feature is the ‘halo’ in between Bragg sheets [marked with arrows ‘3’ in Fig. 2 ▸(*b*)]. The second feature is the ‘shadow’ in between the third and the fourth Bragg sheets [marked with arrows ‘4’ in Fig. 2 ▸(*b*)]. In Section 4 we will show that these ‘halo’ and ‘shadow’ features can be reproduced by simulations taking into account scattering on correlated density fluctuations of the material in the Si layers. Within this approach the position of the ‘shadow’ is due to the size of the density fluctuations and the ‘halo’ is due to the correlation in the positions of fluctuations.

## 3. GISAXS theoretical background   

Calculations of diffuse X-ray scattering are performed using a perturbation theory (Sinha *et al.*, 1988 ▸). A rigorous formulation of the second-order perturbation theory is given in Kaganer *et al.* (1996 ▸). There, the theory is formulated in terms of the reciprocity theorem of electrodynamics (Landau *et al.*, 1984 ▸). In this theorem, the scattering length *f* of the diffuse scattering wave 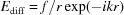 has the form
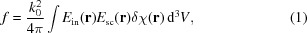
where the function 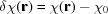 represents a deviation of the actual dielectric susceptibility 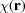 of a structure from the value 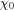 of an ideal structure. Equation (1) is written in a scalar approximation. This 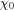 value is used in the calculation of the wavefields in the structure: the field 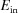 is induced by the incident beam, and the field 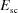 by the diffusely scattered wave. Equation (1) is commonly referred to as the distorted-wave Born approximation (DWBA). We have considered here *s*-polarized radiation, as this was used in the measurements. Therefore, the fields 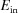 and 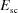 are represented as scalar functions.

The intensity of the diffuse scattering is described with the scattering differential cross section: 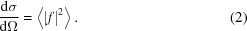
Here, averaging is applied to account for the random nature of imperfections 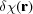 of the structure. 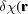) is considered to be a stochastic variable, describing a spatial distribution and structure of the imperfections. In this work we will consider the form of equation (2) explicitly written for interface roughness (in Section 3.1) and the 3D para­crystal of the density fluctuations (in Section 3.2). Finally, the wavefields 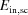 are considered as plane waves with phase terms 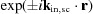. Therefore, considering the integration in equation (1), it is convenient to represent the imperfection of the structure in the form of a Fourier transform: 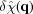, where 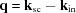. The way of defining the probability density function of 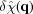 defines a recipe for the simulation of the diffuse scattering on various types of imperfections. We now briefly review the theoretical models.

### 3.1. Scattering on correlated interface roughness   

For the calculation of the diffuse scattering on interface roughness of the multilayer, the wavefield within the *j*th layer is considered to be 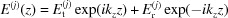. The amplitudes of transmitted 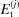 and reflected 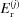 components of the standing wave can be calculated based on a model of the layered structure reconstructed from XRR measurements [see Yakunin *et al.* (2014 ▸) among others]. In the work of Daillant & Bélorgey (1992 ▸), the integration for the scattering length in the DWBA [similar to equation (1)] was done, considering the wavefields described above, resulting in
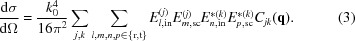
The second summation in equation (3), where the indices 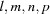 denote the transmitted (t) and reflected (r) waves, has 16 terms. Each term has a correlation function 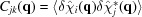 that describes the interface roughness morphology (Pelliccione & Lu, 2008 ▸) of each 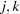 pair of interfaces.

In our numerical simulations, we employ Ming’s model (Ming *et al.*, 1993 ▸) for the calculation. For the characterization of roughness morphology, Ming’s model involves the following parameters: root-mean-square (r.m.s.) roughness amplitude σ, lateral correlation length ξ, Hurst parameter *H* which characterizes the jaggedness of a sample and the vertical correlation length 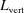. For a detailed description of these para­meters, see Ming *et al.* (1993 ▸) and Siffalovic *et al.* (2011 ▸).

### 3.2. Scattering on the density fluctuations   

Here we consider lateral density fluctuations as an array of spheroids included in a homogeneous matrix of the Si spacer layer. In the STEM image (see Fig. 1 ▸), one can notice that the fluctuations are confined within the Si layers. We note that the vertical sizes are close to the value of the Si layer thickness and are not correlated with the positions along the vertical direction. One can also note that the density fluctuations appear as spheroids of comparable sizes. Additionally, we assume that the density fluctuations are isotropically arranged in the lateral plane inside the Si layers and we neglect correlations between their sizes and positions. Based on these considerations, the density fluctuations are best described using a 3D para­crystal model employing the mono-dispersion and decoupling approximations.

A comprehensive model for the simulation of diffuse scattering on a 3D para­crystal (Eads & Millane, 2001 ▸) is given in Buljan *et al.* (2012 ▸), where scattering from quantum dots is investigated. There, an expression for the differential cross section is derived using a decoupling approximation. In addition to the decoupling approximation, we simplify the expression for the differential cross section given in Buljan *et al.* (2012 ▸) assuming the mono-dispersion approximation: 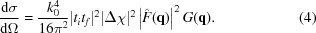
That equation is derived assuming
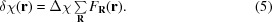
Here 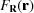 is the shape function of the density fluctuation. It is equal to unity inside the density fluctuation located at 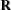 and equal to zero elsewhere.

Taking the Fourier transform of equation (5) in equation (1) results in two functions: 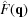 and 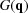. The form factor function 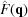 is a Fourier transform of a single density fluctuation shape function 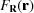 located at the origin 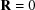. This function is deterministic due to the mono-dispersion approximation. We considered a spheroid shape of the density fluctuation which has a form factor (Lazzari, 2002 ▸) that can be approximated by
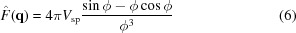
where 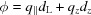; 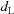 and 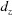 are the lateral diameter and height of the spheroid, respectively, and 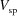 is the spheroid volume.

The correlation function 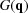 is a sum of phase displacements related to positions of the density fluctuations: 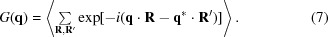
Explicit mathematical expressions for this model are given in Appendix *A*1. The characteristic parameters of this model are: lateral mean distance 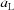 between each two neighbouring density fluctuations, dispersion 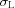 of the lateral mean distance, vertical mean distance 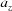 and dispersion 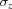 defined analogously to the lateral parameters, lateral 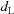 and vertical 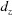 sizes of the density fluctuations. Thus, parameters 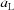, 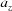, 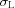, 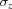 describe positions of the density fluctuations and 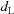, 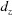 describe the size of the density fluctuations.

## 4. Results and discussion   

### 4.1. Analysis of correlated interface roughness   

**Figure 3:**
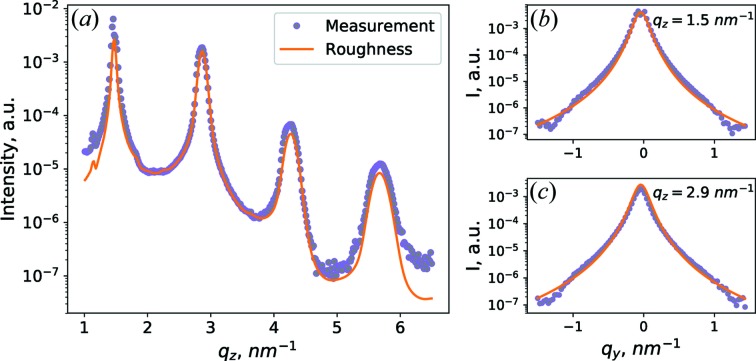
Experimental data (markers) and model simulations based on interface roughness (solid line), for line extractions of data at (*a*) *q_y_* = 0 nm^−1^, (*b*) *q_z_* = 1.5 nm^−1^, (*c*) *q_z_* = 2.9 nm^−1^.

The diffuse scattering due to interface roughness was analysed by fitting model simulations, as described in Section 3.1, to three line extractions of the data: the first line extraction is taken in the plane of incidence (along the *q*_*z*_ direction in *q*_*y*_ = 0 nm^−1^), while the second and third line extractions are in the Bragg sheet planes (along *q*_*y*_ in *q*_*z*_ = 1.5 nm^−1^ and *q*_*z*_ = 2.9 nm^−1^). Experimental data and best fits are shown in Fig. 3 ▸.

The best-fit parameters are: Hurst parameter *H* = 1, roughness r.m.s. amplitude σ = 0.2 nm, vertical correlation length *L*_vert_ = 48.3*D* and lateral correlation length ξ = 8 nm. From the good agreement between simulations of the interface roughness and the experimental data, we conclude that the diffuse scattering along the plane of incidence [Fig. 3 ▸(*a*)] and in the Bragg sheets [Figs. 3 ▸(*b*) and 3 ▸(*c*)] is primarily caused by interface roughness, which is well described by Ming’s model.

The interface roughness at σ = 0.2 nm is considered small. The value of *H* = 1 suggests that interfaces are smooth with no jaggedness. The vertical correlation length (*L*_vert_ = 48.3*D*) is close to the full stack thickness, indicating a high degree of roughness replication from interface to interface.

**Figure 4:**
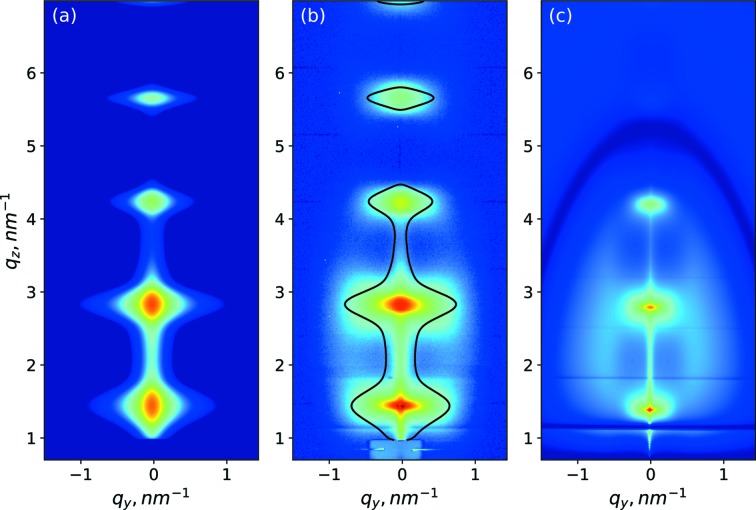
GISAXS (*a*) simulation of scattering from interface roughness, (*b*) experimental data; a black solid contour separates two areas: the inner area is mostly affected by interface roughness; the outer area is mostly affected by density fluctuations. (*c*) Simulation of scattering from density fluctuations inside the Si layers.

The obtained parameters allowed us to simulate the scattering caused by the interfaces for the full range of experimental values of *q_y_*, *q_z_*. The results are shown in Fig. 4 ▸(*a*). As a visual aid, Fig. 4 ▸(*b*) shows the experimental data, together with a contour line based on the simulation of interface roughness scattering that is shown in Fig. 4 ▸(*a*). We attribute the scattering intensity inside this contour line to the correlated interface, and roughness and intensity outside the contour to the density fluctuations.

### 4.2. Analysis of correlated density fluctuations   

The intensity of the scattering from density fluctuations is strongly dependent on the correlation function and the form factor that describe the distribution of the fluctuations. To build the initial model for fitting one can already assume the characteristic parameters of the density fluctuation distribution.

**Figure 5:**
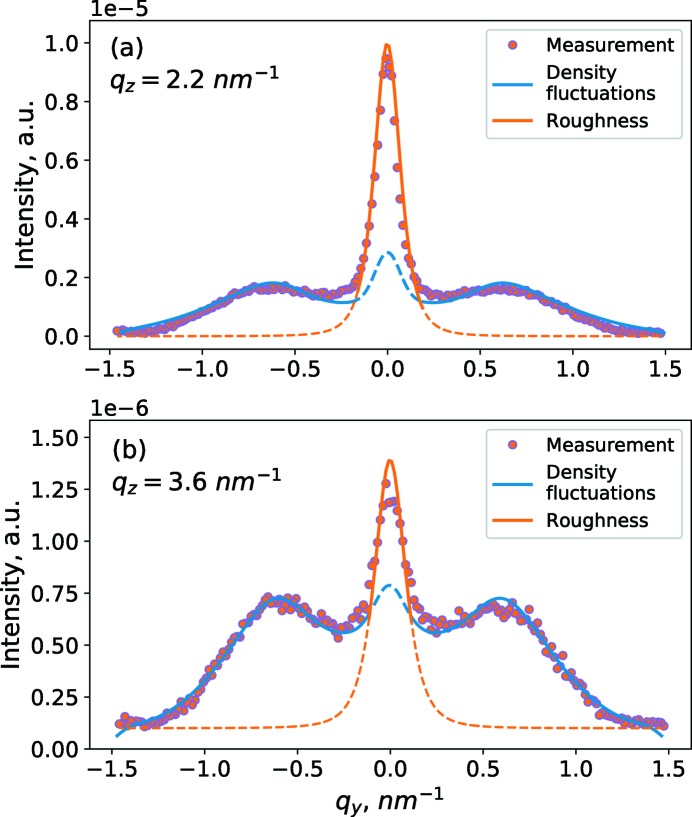
Experimental data (markers) and model simulations based on interface roughness (red line) and density fluctuations (blue line), for line extractions of data at (*a*) *q_z_* = 2.2 nm^−1^, (*b*) *q_z_* = 3.6 nm^−1^.

In the approximation of an ideally ordered distribution of density fluctuations (described in Appendix *A*1), the scattering will have a peak at 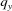 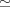 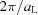. In Fig. 5 ▸ one can see maxima at 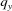 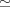 ±0.8 nm^−1^. Thus, as an initial guess we assume that the mean lateral distance 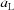 = 8 nm. Tails of the curves in Fig. 5 ▸ are primarily determined by the form factor (see Appendix *A*2). Thus, simulating the slopes of the line-extraction curves in Fig. 5 ▸ (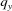 range from 0.9 to 1.5 nm^−1^) as an initial guess, we assume the sizes of the density fluctuations 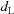 = 4 nm, 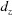 = 2 nm.

Statistical parameters of the density fluctuations were estimated using a best fit to the GISAXS line extractions of experimental data. The confidence intervals of these parameters were estimated using a Hessian matrix calculated at the local minima of the best fit. Statistical parameters of the density fluctuations were also estimated using STEM data (see Appendix *B*1). The results are shown in Table 1 ▸.

Visible in Table 1 ▸ is the excellent agreement between STEM and GISAXS in estimates of the positional parameters (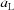, 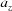, 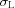 and 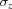). It is noted that the mean lateral distance of the density fluctuations matches very well with the interface roughness lateral correlation 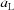 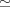 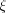 = 8 nm. It hints that formation of the density fluctuations affects interface roughness morphology. The mean vertical distance 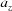 matches very well with the period of the multilayer *D* = 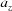 = 4.5 ± 0.2 nm and the dispersion of the mean vertical distance 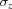 is lower than the r.m.s. roughness amplitude σ estimated by fitting an interface roughness model, 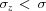 (see Table 1 ▸). Thus, we conclude that the density fluctuations are confined to the Si layer and do not penetrate into the W layer. This observation is consistent with the STEM image shown in Fig. 1 ▸.

Considering the absolute value of the scattered intensity, the size of the density fluctuations and correlated parameters, using equation (4) we find the density contrast: Δρ = 0.26 ± 0.05 g cm^−3^, which is approximately 11% of bulk Si density in normal conditions.

Using the GISAXS best-fit parameters, the intensity of the diffuse scattering was simulated for the full area of the 2D detector shown in Fig. 4 ▸(*c*). In Fig. 4 ▸(*c*) one notices the interesting effect that scattering on the density fluctuations not only causes an enhancement of the scattered intensity between Bragg sheets (‘halo’), but also a ‘shadow’ of the scattering by destructive interference. This ‘shadow’ is defined by the shapes of the density fluctuations in the statistical ensemble. The position of the shadow in Fig. 4 ▸(*c*) is consistent with experimental data in Fig. 4 ▸(*b*). Numerical examples of how ‘shadow’ and ‘halo’ features change with the variation of the parameters of the para­crystal model are discussed in detail in Appendix *A*2. The model used for the numerical simulations is one of the simplest to describe the arrangement of the density fluctuations in the periodical structure. However, the good agreement of simulations with measurement data justifies the approximation used.

Comparing the absolute scattered intensities from interface roughness and density fluctuations, we conclude that diffuse scattering in the studied W/Si multilayer system is primarily due to the interface roughness, with only approximately 8% of the total scattered intensity being caused by the density fluctuations. The formation of the density fluctuations in the Si layers of a W/Si multilayer system is unexpected. Additionally, comparison of the mean lateral distance of the density fluctuations and lateral correlation length of the interface roughness hints that density fluctuations affect the interface roughness morphology. Thus, the analysis of these density fluctuations is of interest for understanding the multilayer growth model.

Our hypothesis is that the density fluctuations in the Si layers are formed due to the interaction with the high-energy back-scattered ions present during the magnetron sputtering deposition. Interacting with the sample surface during the Si layer growth, these high-energy ions distribute energy to the Si layers allowing the formation of the lower-density phase, *i.e.* density fluctuations. Preliminary analysis of various W/Si multilayers deposited with various doses of ion assistance show that higher ion currents result in stronger ‘shadow’ and ‘halo’ effects. For a more detailed investigation of the density fluctuations, the formalism of physical kinetics (Lifshitz *et al.*, 1981 ▸) can be used. That theory allows one to analyse the dynamics of the statistical parameters of density fluctuations. In this article, we estimated the final statistical parameters of the density fluctuations, which can be used in further analysis in the formalism of physical kinetics as a subject of further research.

## 5. Conclusions   

HAADF-STEM and GISAXS were used to study density fluctuations inside Si layers in periodic W/Si multilayers of nanoscale-thickness films. The fluctuations are ordered vertically with a dispersion of 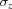 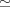 0.11 nm and mean distance 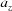 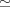 4.5 nm which is equal to the period of the multilayer sample. In the lateral direction, *i.e.* within the Si layer, these density fluctuations have a mean mutual distance of 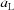 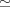 8 nm, while the dispersion in the lateral direction is 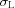 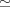 3.2 nm. The density fluctuations are strongly confined within the Si layers and have reduced density (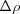 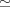 0.26 g cm^−3^). This study exemplifies the level of detail on growth phenomena that can be found using a combination of STEM and GISAXS analysis.

## Figures and Tables

**Figure 6:**
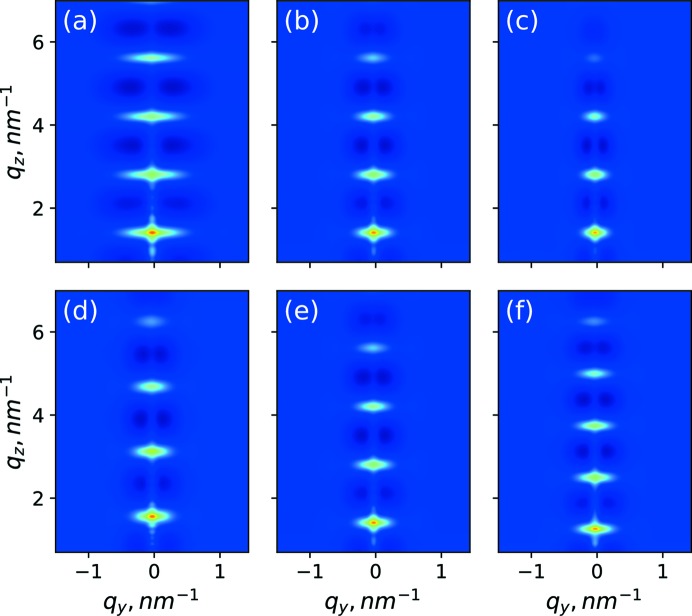
Correlation function 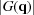 with various values of the mean lateral distance: (*a*) *a*_L_ = 5 nm, (*b*) *a*_L_ = 8 nm, (*c*) *a*_L_ = 12 nm; and with various values of the mean vertical distance: (*d*) *a_z_* = 4 nm, (*e*) *a_z_* = 4.5 nm, (*f*) *a_z_* = 5 nm.

**Figure 7:**
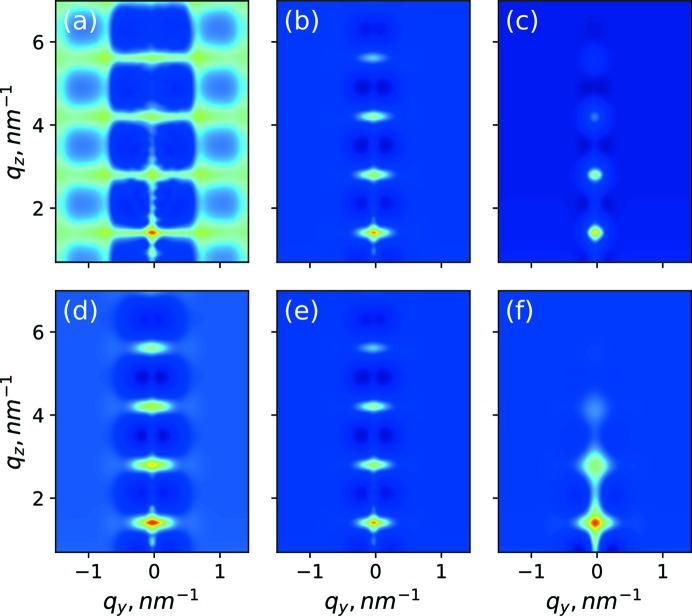
Correlation function 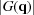 with various values of dispersion in the lateral distance: (*a*) σ_L_ = 0.8 nm, (*b*) σ_L_ = 3.23 nm, (*c*) σ_L_ = 8 nm; and with various values of dispersion in the vertical distance: (*d*) σ_*z*_ = 0.05 nm, (*e*) σ_*z*_ = 0.11 nm, (*f*) σ_*z*_ = 0.5 nm.

**Figure 8:**
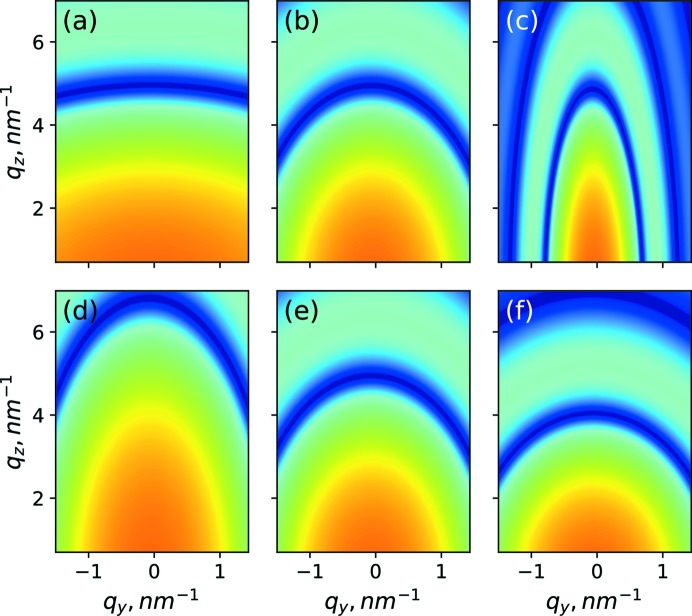
Form factor function 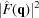 with various values of the lateral size: (*a*) *d*_L_ = 2 nm, (*b*) *d*_L_ = 4.7 nm, (*c*) *d*_L_ = 12 nm; and with various values of the vertical size: (*d*) *d_z_* = 1.3 nm, (*e*) *d_z_* = 1.7 nm, (*f*) *d_z_* = 2.3 nm.

**Figure 9:**
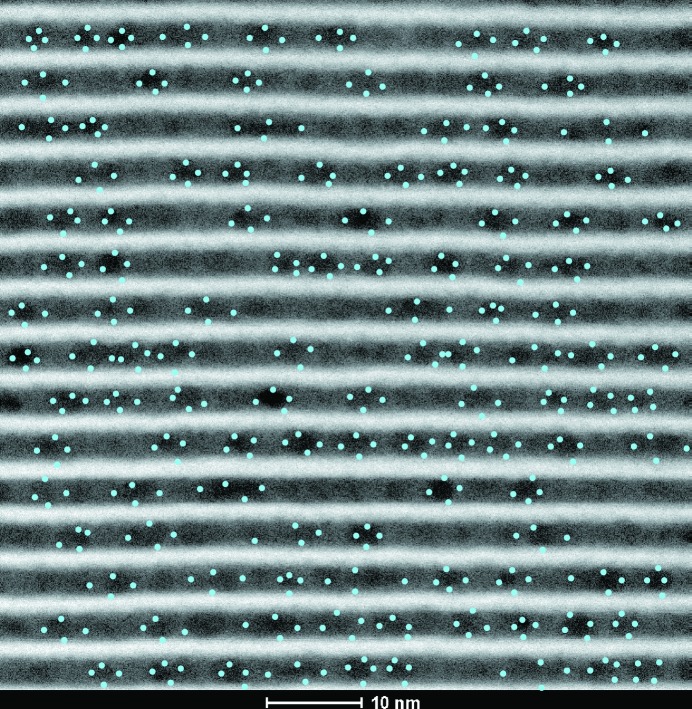
HAADF-STEM image of a W/Si multilayer. Each density fluctuation is manually marked with four dots for the statistical analysis.

**Figure 10:**
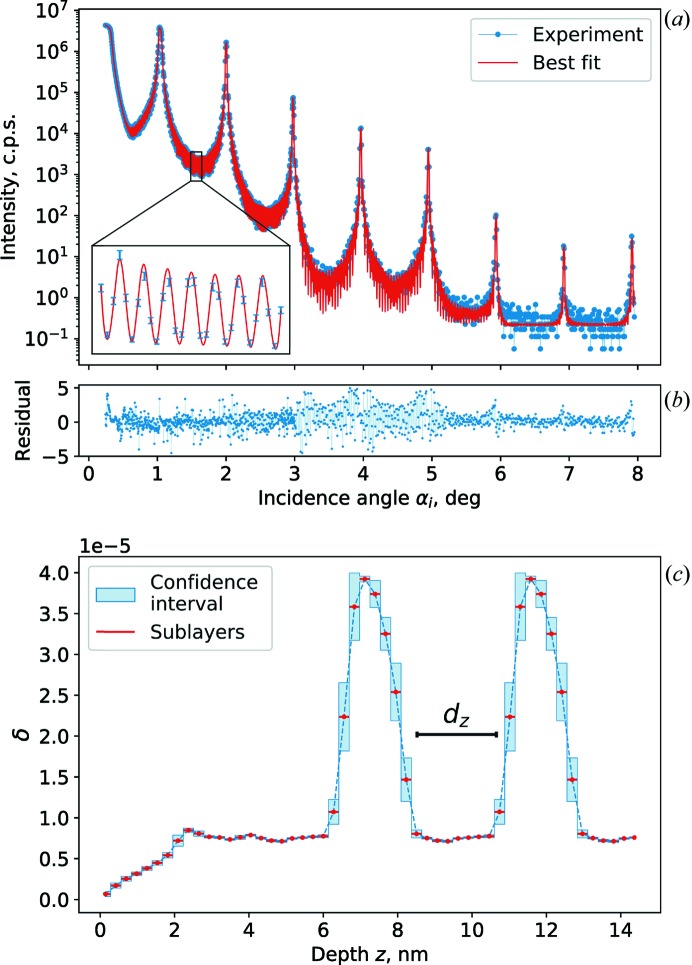
XRR analysis. (*a*) Measured XRR curve and best fit. (*b*) Residuals. (*c*) Best-fit δ profile.

**Table 1:** Calculated parameters of the density fluctuations using HAADF-STEM and GISAXS

	HAADF-STEM	GISAXS
*a*_L_ (nm)	8.1 ± 0.8	8.0 ± 0.6
σ_L_ (nm)	4 ± 1	3.2 ± 0.9
*a_z_* (nm)	4.92 ± 0.09	4.5 ± 0.2
σ_*z*_ (nm)	0.13 ± 0.07	0.11 ± 0.09
*d*_L_ (nm)	4 ± 1	4.7 ± 0.4
*d_z_* (nm)	2.2 ± 0.4	1.7 ± 0.2

## Appendix A Statistical characterization of density fluctuations

### A1. Correlation function of the para­crystal model

For the simulations in Section 4.2, the distribution of the density fluctuations is taken into account using a para­crystal model. Considering the HAADF-STEM data (Fig. 1 ▸) we assume that fluctuations have short-range ordering: the positions are correlated more strongly for neighbouring density fluctuations than for distant density fluctuations. Short-range ordering is taken into account using cumulative position errors (Buljan *et al.*, 2012 ▸). Within that approach the correlation function for a 1D para­crystal lattice has the form

where  =  and τ = ,  =  is the phase displacement related to the para­crystal lattice vector **a**,  =  is attributed to the position dispersion,  is the cumulative position error vector and γ is an effective parameter related to the number of irradiated particles *N* and X-ray absorption:

Assuming that the positions of the density fluctuations are normally distributed, η can be calculated using a general formula (Payne & Clemens, 1993 ▸):

where  is the vector whose components are position dispersion parameters along the *x*, *y* and *z* axes. In the correlation function [equation (8)] absorption of X-rays is taken into account. Note here that equation (8) takes into account 3D displacement of an object from its original positions defined as a 1D para­crystal lattice.

Let us now consider limiting cases. In the case of no absorption, , the correlation function converges to

Both functions, equation (8) and equation (11), have limits:  and . In equation (4) one can notice that dependence of the differential scattering cross section on  is defined with the correlation function and the form factor, equation (6). For  the correlation function is constant. Thus, in this case the scattering intensity is primarily due to the form factor, equation (6). In other words, we can assume that the shape of the scattering ‘tails’ in line extractions of the experimental data shown in Fig. 5 ▸ is solely defined by the form factor. This assumption was used as an initial guess in the fitting procedure in Section 4.2. Finally, in the limiting case of no dispersion , we obtain

In this case the correlation function converges to the interference function. In other words, within the para­crystal model, scattering on an ideal distribution of fluctuations is similar to diffraction from an ideal crystal.

For the numerical simulations in Section 4.2 we approximate the correlation function of the 3D distribution as

where  is the correlation function of the 1D para­crystal model, equation (8), with 3D lattice basis vectors chosen as

and the dispersion is parameterized with vectors:

Strictly speaking, equation (13) does not take into account position cross-correlations between different lattice vectors, *i.e.* displacements of an object along  and , , are considered as statistically independent. To compensate for this in the simulations we performed azimuthal averaging of the correlation function: equation (13) is numerically integrated with respect to rotation of basis vectors around the *z* axis. In the following sections we discuss how variation of the statistical parameters around the best-fit solution affects the correlation function and the form factor.

### A2. Variation of statistical parameters

The correlation function, equation (13), calculated for the best-fit parameters (Table 1 ▸) with various values of the lateral mean distance parameter  is shown in Figs. 6 ▸(*a*), 6 ▸(*b*) and 6 ▸(*c*) and the correlation function with various values of the vertical mean distance  is shown in Figs. 6 ▸(*d*), 6 ▸(*e*) and 6 ▸(*f*).

It has been shown in Appendix *A*1 that for the limiting case  the correlation function converges to a constant value. Indeed, in Fig. 6 ▸ one can see that the correlation function is constant near the edges of the frame. Increasing and decreasing the value of the lateral mean distance  [Figs. 6 ▸(*a*), 6 ▸(*b*) and 6 ▸(*c*)] results in a change of the ‘halo’ along the  axis. The width of the ‘halo’ is proportional to . A change of the vertical mean distance  [Figs. 6 ▸(*d*), 6 ▸(*e*) and 6 ▸(*f*)] results in a shift of Bragg sheets and the ‘halo’ along the  direction. It is important to note here that Bragg sheets in Figs. 6 ▸(*d*), 6 ▸(*e*) and 6 ▸(*f*) are not caused by interface roughness. Here, Bragg sheets are due to the periodicity of the density fluctuations in the *z* direction. In the experimental data in Fig. 4 ▸(*a*) these peaks are not resolvable due to the interface roughness contribution to the diffuse scattering [Fig. 4 ▸(*b*)]. However, the vertical position of fluctuations can be estimated by the shape of the ‘halo’ feature.

In the HAADF-STEM image (Fig. 1 ▸) one can see that density fluctuations are confined within the Si spacer layer. The same conclusion can be drawn from best-fit parameters: mean vertical distance  is equal to the period of the multilayer  4.5 nm and dispersion of the mean vertical distance  is lower than the r.m.s. roughness amplitude σ estimated by fitting the interface roughness model, . Let us consider the case in which fluctuations start to penetrate the W layer. One can simulate that situation by increasing parameter . In that case the ‘halo’ features are blurred significantly already at the dispersion values of  0.5 nm [see Fig. 7 ▸(*f*)].

In the opposite case of low values of distance dispersion  = 0.11 nm and  = 0.8 nm [see Fig. 7 ▸(*a*)] the correlation function is similar to the diffraction on the crystal powder. That observation is consistent with equation (12) where the limiting case  is considered.

The form factor function, equation (6), calculated for various size parameters is shown in Fig. 8 ▸. One can observe in Fig. 8 ▸ that the position of the ‘shadow’ from the best-fit model [see Fig. 4 ▸(*c*)] is not as broad as it is in the experimental data [see Fig. 4 ▸(*a*)]. This might be due to the mono-dispersion approximation. Finally, one can observe in Fig. 8 ▸ that the value of the form factor decreases along the  direction. Since the scattering differential cross section distribution along  is defined only with the correlation function and the form factor [see equation (4)] and the correlation function is constant near the edge of the frame in Fig. 6 ▸, the tails in Fig. 5 ▸ are defined by the form factor.

## Appendix B Details of preliminary sample characterization

### b1. Statistical analysis of HAADF-STEM image

In this section we describe the procedure that has been employed for estimation of the statistical parameters in Table 1 ▸ from the STEM image. The boundaries of fluctuations were manually marked on the STEM image, four points for each dark object in Fig. 9 ▸. The position of each fluctuation has been calculated as a centre of mass of these four points. Lateral and vertical sizes have been calculated for each fluctuation as  and , respectively, where . Thus statistical parameters have been estimated. To estimate confidence intervals of these parameters we used a *t* distribution. Position parameters , ,  and  were considered to be normally distributed, while size parameters  and  were considered to be gamma-distributed, since variation of size parameters is large and negative values are nonphysical. Negative  and  parameters are nonphysical as well; however, since variation of these parameters is smaller than that of the size parameters, we therefore consider a normal distribution to be a valid assumption for these parameters.

### b2. XRR analysis

XRR curve fitting has been used for a preliminary characterization of the sample. XRR was measured on a laboratory diffractometer using the characteristic Cu *K*α radiation (incident-beam wavelength λ = 0.154 nm). The free-form approach was used for XRR fitting [see Zameshin *et al.* (2016 ▸) for a detailed description]. The XRR curve was measured up until the incidence angle  = 8°. That corresponds to the maximal vertical momentum transfer  = 11.4 nm^−1^. According to the sampling theorem, the minimal discretization step of the optical constant profile (δ profile) is *d*_min_ = . Fitting by a model with a discretization step lower than *d*_min_ does not provide relevant information about the structure of the sample. Therefore, the model of the W/Si bilayer period contained 16 sublayers with individual thickness of approximately 0.28 nm each. In the model all 50 periods were assumed to be identical. The measured XRR curve was fitted by a simulated curve using the derivative-free bound-constrained optimization algorithm BOBYGA (Powell, 2009 ▸). The  functional (Hughes & Hase, 2010 ▸) was used as a goodness-of-fit criterion. The measured XRR curve and the best-fit simulated curve are shown in Fig. 10 ▸(*a*). Residuals normalized on measurement uncertainty are shown in Fig. 10 ▸(*b*). The best-fit model corresponds to the goodness-of-fit criterion of  = 2.3. The δ profile of the best fit is shown in Fig. 10 ▸(*c*). Model uncertainties [blue error corridors in Fig. 10 ▸(*c*)] were calculated using the formalism of an inverse Hessian.

In the model, the surface of the sample was considered separately from the period to take oxidation into account. That allowed us to improve the goodness of fit in between Bragg peaks [see the inset in Fig. 10 ▸(*a*)]. As a reference, the vertical size of the density fluctuations *d_z_* is shown in Fig. 10 ▸(*a*). One can notice that the vertical size of the density fluctuation fits perfectly in the Si layer, which is consistent with GISAXS and TEM data.
